# Disentangling food transformation narratives: a Q-method analysis on pathways to sustainable food systems and their implications

**DOI:** 10.3389/fnut.2025.1662085

**Published:** 2025-09-10

**Authors:** Meredith L. Mull, Mario Torralba

**Affiliations:** Institute for Environmental Studies (IVM), Vrije Universiteit Amsterdam, Amsterdam, Netherlands

**Keywords:** food system transformation, sustainable food systems, pathways to sustainability, SDG 12, food systems

## Abstract

**Introduction:**

While the urgency to transform global food systems is widely recognized in scientific and policy circles, differing interpretations of what constitutes a sustainable food system continue to challenge coordinated action. Understanding these diverse perspectives is essential for designing inclusive and effective transformation pathways.

**Methods:**

This exploratory study used Q-methodology to investigate how university students enrolled in sustainability-related programs conceptualize sustainable food systems. Participants sorted and ranked statements reflecting various food system priorities, enabling the identification of shared and divergent viewpoints.

**Results:**

Analysis revealed five distinct narratives: (1) securing food sovereignty, (2) contributing to climate justice, (3) doing no harm, (4) empowering consumers, and (5) connecting people to their food. Despite these differences, consensus emerged around the importance of food security and transparent, democratic governance. In contrast, elements such as urban agriculture, GMOs, and the preservation of food traditions were consistently deprioritized.

**Discussion:**

The findings highlight the value of incorporating plural perspectives into food system research and policy. Consensus elements may serve as bridging concepts to foster dialogue and collaboration across diverse stakeholder groups. The deprioritization of certain themes underscores the need for context-sensitive approaches that reflect the lived experiences and priorities of specific populations.

## Introduction

Both scientific and policy agendas urgently recognize the need to transform the food system, emphasizing the interdependence of human and planetary health (1–3). Globally, the food system plays a pivotal role across multiple sustainability dimensions: environmentally, as a major contributor to the crossing of several planetary boundaries (4); economically, substantially contributing to the GDP in many countries, although its benefits are increasingly concentrated in the hands of a few powerful actors (5); and socially, supporting livelihoods of more than two billion people (6), yet still failing to provide sufficient food for 820 million people (3).

While the need for food system transformation is agreed upon, considerable variation exists in what this transformation would entail among food system actors. The concept of a sustainable food system currently comprises a broad range of concepts, approaches, and principles (7–9). These may be similar, but they can also be different or even contradictory. In practice, what sustainable food system transformations mean for a specific context can greatly differ, depending on a region’s needs and the assortment of values, backgrounds, and personal contexts represented in its population. Different interpretations have varied implications for environmental governance and impacts, and understanding these distinctions is critical to instigating transformations at any level or scale.

Food systems encompass the interactions within a social-ecological system that are associated with the production, processing, distribution, and consumption of food, with the primary goal of ensuring food security (10, 11). While food security is primary, achieving it sustainably necessitates considering three dimensions – ecological, social, and economic – that inherently have multidimensional variations in their aims and outcomes, which can sometimes come at the expense of each other (12). Sustainable food systems support the achievement of food security and nutrition while also supporting the social-ecological systems on which they rely. Managing natural resources within agroecosystems, ensuring food security, and guaranteeing prosperous livelihoods along the food value chain all fall within the scope of sustainable food systems. Some authors, such as Swinburn et al. (2), incorporate these aspects into their definition of a sustainable food system and additionally highlight global outcomes of social equity and economic prosperity. Furthermore, the United Nations’ Sustainable Development Goals (SDGs) are directly related to sustainable food system outcomes through SDG 12, but also indirectly through SDGs 2, 3, 8, 13, 14 and 15 (9). A recent progress report suggests that substantial stagnation and regression have occurred across all goals, and that transforming the food system remains essential for developing a just and sustainable future (13).

There are multiple, and often contested, targets and pathways proposed for achieving sustainable food systems. However, the myriad of economic and political interests at play within global food systems makes their current status a paradigmatic example of wicked problems. The transformation of food systems is, in itself, a rebuke of the status quo where certain interests have disproportionate influence over decisions (1). The rebuke of the economic and socio-political dimensions underpinning global food systems is for some authors a prerequisite for their transformation, where a level playing field is a priority to address the power imbalances of the current food system and to foster food sovereignty (7, 14, 15).

The complex, intersecting dimensions of sustainable food systems encompass a wide range of perspectives driven by varied interests but ultimately require a collective effort that involves all sectors of society. These perspectives have been significantly shaped by academic research and various food movements, particularly those focused on food justice and the environmental impacts of industrial food systems (16, 17). This body of work emphasizes systemic issues, such as racial and economic disparities, and pushes for food systems that are inclusive, equitable, and sustainable for marginalized communities (18).

Society relies on food for biological sustenance, but also relates to food through a range of personal, cultural, ideological, or societal factors, which need to be taken into account for the transformation of food systems (19, 20). Therefore, understanding what drives each perspective and how those underlying preferences, values, and narratives are part of the whole, is a key component to analyzing potential pathways for transformation.

By exploring how narratives of sustainable food systems are perceived, the main objective of this study is to identify the diverse viewpoints and priorities regarding what characterizes a sustainable food system, and where these perspectives converge or diverge. Our study focuses on university students enrolled in sustainability-related programs, considering them a crucial generation of future professionals poised to advance food system sustainability and uniquely positioned to bridge theoretical knowledge with practical application. To do so, we employ Q-sort interviews to uncover elements of consensus and potential frictions within possible pathways for food system transformation.

## Materials and methods

Q-sort interviews or Q-method is a methodology that enables both in-depth qualitative and quantitative statistical interpretations of human subjectivity using a multiple-participant format to explore complex and even contested concepts from participants’ viewpoints (21–23). The Q-method explores narratives that emerge from participants’ responses, in our case specifically to the ranking of statements that evoke values and interests related to sustainable food systems. This configuration of ranked items by each participant, known as a factor array, is the basis of the statistical analysis.

In our Q-method interviews, the guiding question for the ranking was, “What are the characteristics of a sustainable food system?” To answer this, we provided respondents with a set of 37 statements that were developed through an iterative process. We began by inductively extracting a comprehensive list of concepts related to food system sustainability from a wide range of academic scholarship and international reports, which were iteratively refined. Through a process of merging similar concepts and splitting complex ones, we selected and formulated each statement to represent a single, distinct component associated with food system sustainability (Supplementary material 1).

**Figure 1:**
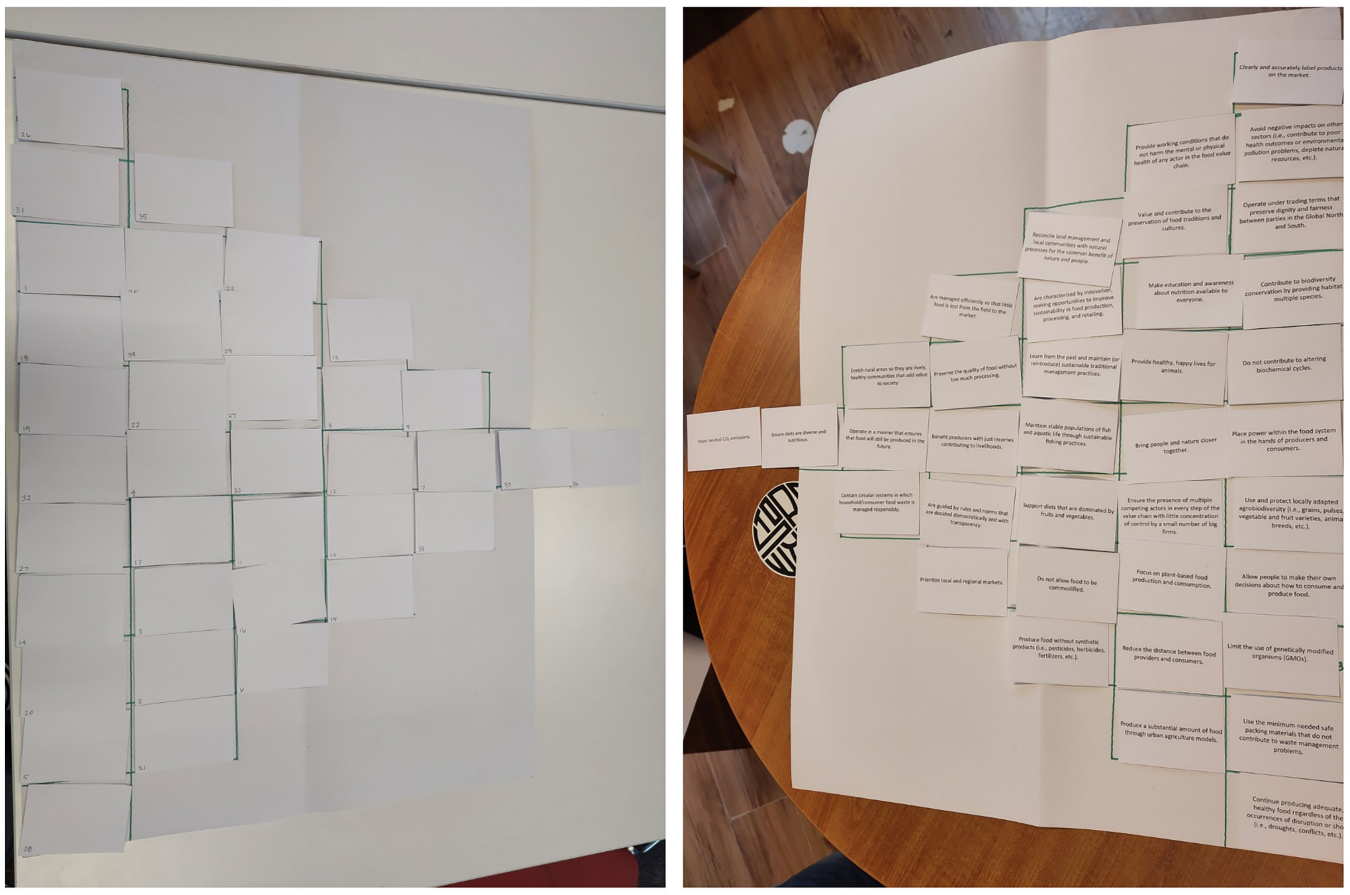
Q-sorts completed on posterboard ranking grid in forced quasi-normal distribution with a set of 37 cards.

Collectively, the statements synthesized the range of core guiding principles defining food system sustainability, such as concepts underpinning food security, SDGs with implications for the food system, the concept of resilience, and the diverse principles pursued by international programs, such as WHO and FAO. Participants were asked to allocate the statements from the Q-set on a ranking grid based on how much each statement represented, related to, and connected with their own narrative of a sustainable food system (on a scale of “most important” to “least important”), as shown in Figure 1. The sorting activity was first piloted with a small group of participants to refine the Q-set and finalize the ranking grid before data collection. The ranking grid was arranged in a forced quasi-normal distribution. After ranking the statements, participants completed a short, open-question survey to contextualize their responses. This supported the interpretation of the results and allowed participants to explain their reasoning for assigning the most extreme values.

We employed a random, purposive sampling strategy among the (Removed to preserve the anonymity of the authors) student population enrolled in sustainability-related bachelor, master, or PhD programs (Supplementary material 2). A total of 31 participants were interviewed. Participants were approached directly in public spaces on campus, as well as through online communication channels for various student groups, study programs, and extracurricular activities. The participants comprised primarily university students, predominantly aged 18–34, with a mix of genders and diverse cultural backgrounds and enrolled in sustainability-related programs indicating a pre-existing interest in the study’s subject, though some come from fields like Chemistry or Data Sciences. The interviews were collected in April 2024 on campus using two sets of posterboards and cards on which participants could physically sort the Q-statements (Figure 1). Participants could also carry out the survey using the software Q-sortware (24). It took participants around 25 min to complete the sorting activity and respond to the post-sorting interview questions. Our application of the Q-method had some limitations that need to be considered for the interpretation of the results, including a potential for demographic bias in our participant sample, and a possible self-selection bias in the recruitment strategy. Furthermore, the international setting of the university, where English is a primary language for learning but may be a non-native language for most participants, may have limited the expression of personal narratives to some degree. While our intention in this study is to explore how narratives of sustainable food systems are expressed and perceived, and use university students as a case study, to address this potential bias, it would be a valuable research pathway to explore how different demographics perceive and prioritize sustainable food system transformations, helping to broaden our study’s methodological approach.

### Data analysis

To discriminate between the different narratives within our sample, we used a principal component analysis with varimax rotation. We carried out the analysis using the qmethod R package (25). We employed various criteria to decide the number of factors to extract and finally selected five factors. We first looked at the five-factor configurations and used factor scores to reconstruct the ranking grid for each of them. A sort needed to load at 0.32 in each factor to meet a 0.095 significance threshold, as calculated by alpha = 0.05, where the number of statements was 37. The five-factor configuration captured distinct viewpoints that were not otherwise observed, with factor correlations showing acceptable differentiation (41% similarity in the 5-factor extraction compared to 36% similarity in the 2-factor). All eigenvalues were greater than one, meeting the Kaiser-Guttman criteria, and the cross-product of the two highest factor loadings exceeded 0.01, satisfying Humphrey’s rule. Considering these criteria, we chose to interpret the analysis with five factors.

## Results

**Figure 2:**
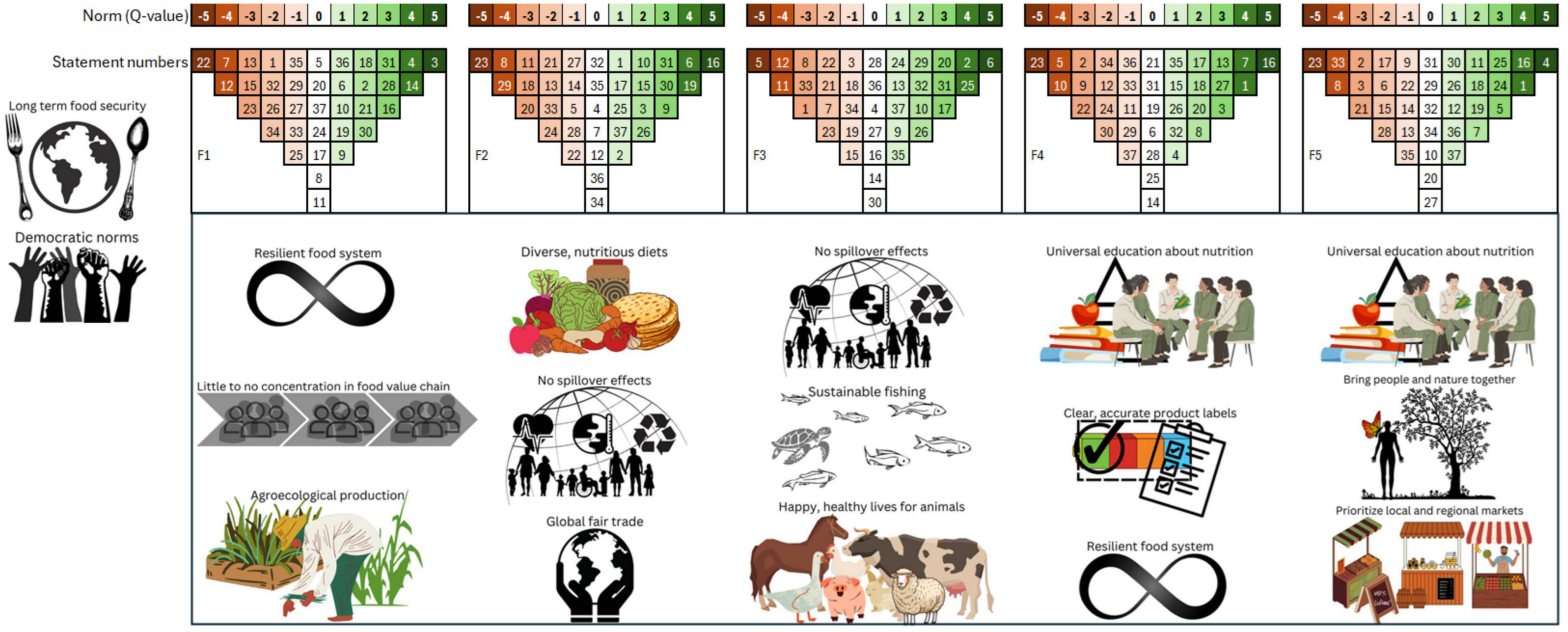
For each narrative, the figure includes ranking grids and graphics highlighting characteristics with the highest z-score values. These grids display an average of each Q-set statement’s relative normative value, derived from Q-sorts loaded onto that specific factor. Numbers within the cells refer to statement numbers in Table 1, with statements possessing the highest weighted z-score values positioned at the grid’s periphery. Directly beneath each factor’s grid, graphics illustrate the most highly valued characteristics of sustainable food systems within that narrative. Additionally, graphics to the left of the figure depict the overall most valued characteristics identified in the entire study.

The Q-sort analysis yielded five factors (Table 1; Figure 2), each representing a unique narrative of sustainable food system transformation. These narratives, each with it own specific focus and priorities, are: (1) sustainable food systems secure food sovereignty; (2) sustainable food systems contribute to climate justice; (3) sustainable food systems do no harm; (4) sustainable food systems empower consumers; (5) sustainable food systems connect people to their food.

**Table 1:** Factor numerical representations: for each statement in the Q-set, the z-score and normalized Q-score are provided across the five sustainable food system transformation narratives.

Statement		F1 Secure food sovereignty		F2 Contribute to climate justice		F3 Do no harm		F4 Empower consumers		F5 Connect people to their food		Dist./Cons./relevance
Sustainable food systems		Norm	*Z*	Norm	*Z*	Norm	*Z*	Norm	*Z*	Norm	*Z*	
1.	Make education and awareness about nutrition available to everyone.	−2	−0.76	1	0.18	−3	−1.05	4	1.44	4	2.32	F2, F4, F5
2.	Provide healthy, happy lives for animals.	2	0.81	1	0.63	4	1.40	−3	−1.51	−3	−1.41	F3
3.	Continue producing sufficient, nutritious food regardless of disruptions or shocks (i.e., droughts, conflicts, etc.).	5	1.93	2	0.90	−1	−0.54	3	1.12	−3	−1.32	F1, F3, F5
4.	Are guided by rules and norms that are decided democratically and with transparency.	4	1.51	0	−0.15	0	0.00	1	0.57	5	2.88	F1, F5+
5.	Bring people and nature closer together.	0	−0.32	−1	−0.51	−5	−1.98	−4	−1.84	3	0.93	F5
6.	Avoid negative impacts on other sectors (i.e., contribute to poor health outcomes or environmental pollution problems, deplete natural resources, etc.).	1	0.39	4	1.60	5	1.86	0	0.14	−2	−0.82	F5
7.	Clearly and accurately label products on the market.	−4	−1.43	0	−0.15	−2	−0.83	4	1.25	2	0.76	F1, F2, F3
8.	Allow people to make their own decisions about how to consume and produce food.	0	0.04	−4	−1.24	−3	−1.58	2	0.87	−4	−1.5	F1, F4
9.	Operate under trading terms that preserve dignity and fairness between parties in the Global North and South.	1	0.67	3	1.30	1	0.42	−3	−1.35	−1	−0.62	F2, F4, F5
10.	Focus on plant-based food production and consumption.	1	0.39	2	0.73	2	0.94	−4	−1.55	0	0.10	F4
11.	Do not allow food to be commodified.	0	0.18	−3	−1.16	−4	−1.74	−1	−0.20	2	0.53	F2, F3
12.	Enrich rural areas so they are lively, healthy communities that add value to society.	−4	−1.39	0	−0.08	−4	−1.86	−2	−1.00	1	0.34	
13.	Learn from the past and maintain (or reintroduce) sustainable traditional management practices.	−3	−1.33	−2	−0.85	1	0.36	3	0.93	−1	−0.26	
14.	Ensure the presence of multiple competing actors in every step of the value chain with little concentration of control by a small number of big firms.	4	1.64	−1	−0.67	0	0.20	0	0.33	−1	−0.43	F1
15.	Are characterized by innovation, seeking opportunities to improve sustainability in food production, processing, and retailing.	−3	−1.31	2	0.90	−1	−0.19	1	0.37	−2	−0.71	
16.	Operate in a manner that ensures that food will still be produced in the future.	3	1.42	5	2.11	0	0.13	5	2.27	4	1.18	F3+
17.	Are managed efficiently so that little food is lost from the field to the market.	0	0.00	1	0.33	3	1.36	2	0.68	−2	−0.91	F3, F5
18.	Use and protect locally adapted agrobiodiversity (i.e., grains, pulses, vegetable and fruit varieties, animal breeds, etc.).	2	0.80	−3	−1.01	−1	−0.35	2	0.80	2	0.66	F2, F3
19.	Ensure diets are diverse and nutritious.	1	0.44	4	1.89	−1	−0.26	0	0.12	2	0.74	F2
20.	Reduce the distance between food providers and consumers.	0	−0.08	−3	−0.97	3	1.11	2	0.83	0	0.12	F2
21.	Place power within the food system in the hands of producers and consumers.	2	0.92	−2	−0.87	−2	−0.90	0	−0.13	−3	−0.95	F1, F4
22.	Produce a substantial amount of food through urban agriculture models.	−5	−2.31	−1	−0.35	−2	−0.90	−3	−1.26	−1	−0.47	F1−
23.	Limit the use of genetically modified organisms (GMOs).	−3	−1.31	−5	−2.60	−2	−0.58	−5	−2.13	−5	−1.77	F3−
24.	Prioritize local and regional markets.	0	0.00	−2	−0.76	1	0.25	−2	−0.99	3	0.88	
25.	Maintain stable populations of fish and aquatic life through sustainable fishing practices.	−1	−0.42	1	0.51	4	1.59	0	0.30	3	0.85	F1, F3
26.	Have neutral CO_2_ emissions.	−2	−0.65	2	1.01	2	1.11	1	0.45	1	0.25	F1
27.	Preserve the quality of food without too much processing.	−1	−0.52	−1	−0.67	0	0.08	3	1.12	0	0.21	F4
28.	Reconcile land management and local communities with natural processes for the common benefit of nature and people.	3	1.21	−1	−0.37	0	−0.03	0	0.21	−2	−0.71	F1
29.	Produce food without synthetic products (i.e., pesticides, herbicides, fertilizers, etc.).	−1	−0.63	−4	−1.16	2	0.61	−1	−0.18	0	−0.03	F3
30.	Benefit producers with just incomes contributing to livelihoods.	2	1.07	3	1.09	0	0.23	−2	−0.42	1	0.22	F4
31.	Provide working conditions that do not harm the mental or physical health of any actor in the food value chain.	3	1.18	3	1.04	3	1.18	0	0.00	0	−0.04	
32.	Use the minimum needed safe packing materials that do not contribute to waste management problems.	−2	−0.74	0	−0.30	2	0.85	1	0.49	0	−0.03	
33.	Value and contribute to the preservation of food traditions and cultures.	−1	−0.45	−2	−0.78	−3	−1.48	−1	−0.37	−4	−1.62	−
34.	Support diets that are dominated by fruits and vegetables.	−2	−0.64	0	0.06	−1	−0.32	−2	−1.17	0	−0.02	
35.	Contain circular systems in which household/consumer food waste is managed responsibly.	−1	−0.63	0	−0.19	1	0.51	1	0.35	−1	−0.25	
36.	Do not contribute to altering biogeochemical cycles (e.g. carbon, nitrogen, water).	1	0.36	0	0.05	0	0.00	−1	−0.40	1	0.38	Cons.
37.	Contribute to biodiversity conservation by providing habitat for multiple species.	0	−0.02	1	0.53	1	0.38	−1	−0.13	1	0.49	Cons.

The table’s order reflects distinctiveness, with the most distinctive statements (based on z-score difference) at the top and consensus statements at the bottom (labeled “Cons.”). Distinguishing statements (at *p* < 0.05) indicate which factor they differentiate. A “+” sign denotes statements considered relevant to all perspectives, while a “−” sign indicates those considered irrelevant by any perspective.

### Narrative 1: secure food sovereignty

The first narrative, shared by six participants, centers on resilience as critical component of sustainable food systems, emphasizing diversity, fairness, and democratic processes and structures. The core of this perspective lies in the concepts of food sovereignty and agroecology, aiming to empower producers and consumers. For example, within this narrative, ensuring multiple, competing actors at every step of the value chain (Statement 14) is a key driver for food system transformations. Interviews further supported this, with one respondent highlighting the importance of actor diversity due to its “*potential cascading effects and ability to change the structure of the global food system*” (Participant 2). This is further indicated by the statement, “*If the concentration of power in the value chain is eliminated, the rest of the food system transformation would be much easier to implement or might even come intuitively*” (Participant 1). This perspective frames food as a human right and a matter of international law, with transformation strategically driven by breaking up institutional and corporate dominance.

An important distinguishing factor associated to this narrative is the importance of a continued sufficient food production even in the face of potential disruptions or shocks (Statement 3). This is illustrated by responses such as “*a stable supply of food, even in war, conflict, climate change, and market failures, is critical*” (Participant 29). According to this perspective, the food system should be guided by rules and norms decided transparently and democratically (Statement 4). Furthermore, producing food agroecologically is vital to this narrative, as evidenced by the importance of two characteristics: using and protecting locally adapted agrobiodiversity (Statement 18) and reconciling land management and local communities with natural processes for the common benefit of nature and people (Statement 28). This is supported by comments such as “*Land use change is the main driver of biodiversity decline, and the lack of a holistic governance exacerbates that. So, I find land management practices to be the most important characteristic to address*” (Participant 5).

### Narrative 2: contribute to climate justice

Narrative 2, shared by seven participants, is characterized by a strong emphasis on fairness and justice, seeking to repair the legacies of unjust historical dynamics of the global food system. This perspective highlights how the food system must operate under trading terms that preserve dignity for Global North and Global South actors (Statement 9) and emphasizes the quality of life and work of people within the food system, including just incomes (Statement 30) and working conditions that do not harm workers’ mental or physical health (Statement 31). This food system transformation pathway also focuses on reducing negative impacts on other sectors (Statement 6). Innovation is viewed as a key driver of this transformation, aiming to improve sustainability at each stage of the food value chain (Statement 15). In parallel, from this perspective, diverse and nutritious diets (Statement 19) that are dominated by fruits and vegetables (Statement 34) become the norm, food is fairly traded, and the food system is carbon neutral (Statement 26).

Ensuring equal access to high quality food that will still be produced in the future (Statement 16) distinguishes this narrative the most, and, from this perspective, “other issues can be sorted out later” (Participant 4). Plant-based food production and consumption (Statement 10) is important to this narrative of sustainable food system transformations; thus, addressing the land use change aspect of the current unsustainability of the food system. Whereas global trends toward increased animal product consumption present food systems with a critical land use challenge, this narrative appreciates the importance of using land to grow food for direct consumption by humans.

### Narrative 3: do no harm

Narrative 3, shared by five participants, aims to eliminate any harm to people, to animals, or to the environment. Avoiding negative impacts and spillover effects (Statement 6) is indicated by respondents to be “*the most all-encompassing characteristic of a sustainable food system*” (Participant 24). Progress toward the SDGs is most apparent in this narrative. This entails, among others, prioritizing contributing to biodiversity conservation by providing habitat for multiple species (Statement 37), not contributing to altering biogeochemical cycles (Statement 36), and maintaining stable populations of fish and aquatic life through sustainable fishing practices (Statement 25). The carbon neutrality (Statement 26) of the system is emphasized, as indicated in statements such as, “*at the core, sustainable initiatives should be carbon neutral in order to remain within the planetary boundaries and to avoid negative feedback loops that could occur beyond certain thresholds*” (Participant 18). Additionally, adherence to the Lancet Commission recommendations, including halving food waste and loss by 2030, halving animal product consumption, and doubling fruit and vegetable consumption, are results of this food system transformation narrative. Food production systems from this perspective are organic, non-GMO, plant-based, circular, and local.

Resource and waste management are also central to this transformation pathway, as evidenced by the importance of three characteristics: using the minimum needed safe packing materials that do not contribute to waste management problems (Statement 32); efficient management so that little food is lost from the field to the market (Statement 17); and circular systems in which household/consumer food waste is managed responsibly (Statement 35). This narrative is grounded on ethical considerations and desires a responsible and precautionary approach. Animal welfare (Statement 2) is a priority in this narrative, along with human welfare (Statement 31). Together these important qualities characterize this food system transformation as being protective of all human and natural resources.

### Narrative 4: empower consumers

Narrative 4, shared by six participants, is characterized by challenging current norms and rules in the processing and retail sectors. It argues that everyone deserves education and awareness about nutrition (Statement 1), and products should be clearly and accurately labeled (Statement 7). Furthermore, access to this information would empower people to make their own decisions about how to consume and produce food responsibly (Statement 8). Primarily, the prospect of long-term food security (Statement 16) is important to this narrative, as is the capacity to withstand shocks (Statement 3); and there is concern that “*population growth may lead to a global food shortage*” (Participant 6). Driving this food system transformation are lessons from the past about how to manage environmental resources through sustainable and traditional practices.

Two key characteristics of sustainable food systems prompted contextual data from research participants about the personal nature they held for individuals in the food system: preserving the quality of food without too much processing (Statement 27) and clearly and accurately labeling products on the market (Statement 7). For the individual consumer, food preferences are seen to be compromised without changes to these aspects of the food system. This is indicated by statements like, “*As consumers, we face confusion when making choices about what to buy when faced with complex ingredients and inaccurate labels*” and that nutrition or sustainability goals “*are at stake when it comes to making sense of unclear labels*” (Participant 13). One participant also noted “*the frustration of spending time in the supermarket aisle reading a package and then returning home to see unexpected information on the packaging that was not noticed before purchasing*” (Participant 9). Both characteristics are highly pronounced in this narrative.

Significantly, this narrative is less concerned with an orientation toward plant-based production and consumption (Statement 10), diets dominated by fruits and vegetables (Statement 34), animal welfare (Statement 2), sustainable fishing (Statement 25), or contributing to biodiversity conservation (Statement 37). However, it places higher value on the preservation of food tradition and cultures (Statement 33) and learning from traditional food systems (Statement 13). The so-called “middle spaces” of the food system are held to account, and there is a power shift in this transformation that limits too much interference by dominant actors in the processing and retail sectors.

### Narrative 5: connect people to their food

The fifth narrative, shared by four participants, envisions communities closely connected to their food source. Bringing nature and people together (Statement 5) is central for food system transformation. This is indicated by responses suggesting that an “*overarching quality of food systems being people close to nature would cause other sustainable characteristics to become true as well*” (Participant 16), and that people being close to nature is a form of “*resistance to the current capitalist paradigm*” (Participant 31). Consequently, using and protecting locally adapted agrobiodiversity (Statement 18) and contributing to biodiversity conservation by providing habitat for multiple species (Statement 37) are considered highly important. Moreover, local and regional markets are prioritized (Statement 24), and commodified food is rejected in food systems of the future (Statement 11). Most distinctive in this narrative is that rules and norms regarding the functioning of the food system should be decided transparently and democratically (Statement 4), lending to the transformation being driven by committed political will.

A close relationship between humans and the environment is expected to lead toward healthy land and water ecosystems. Knowledge about sustainable food systems is generated through personal contact with the natural systems where food production occurs, and it follows that more emphasis is placed on enriching rural areas, so they are lively, healthy communities that add value to society (Statement 12). Furthermore, with this knowledge, both production and consumption trends improve: food production uses fewer synthetic products (Statement 29), biogeochemical cycles are not disrupted (Statement 36), and agriculture is less reliant on big machinery, producing fewer CO_2_ emissions (Statement 26). Similarly, stable fish and aquatic populations are maintained (Statement 25), and biodiversity through habitat preservation (Statement 37) as well as agrobiodiversity are protected (Statement 18). Diets that are diverse, nutritious, and dominated by fruits and vegetables (Statements 19, 34) are widely adopted. Together, these qualities characterize this narrative as connecting people to their food.

### Similarities and differences across narratives

The five identified narratives shared some characteristics (Table 1). There were two core principles that were positively considered as a priority in sustainable food systems for all the identified narratives: operating in a manner that ensures food will still be produced in the future (Statement 16) and being guided by rules and norms that are decided democratically and with transparency (Statement 4). Ensuring long-term food security is highly important and even essential to all narratives, as indicated by statements like, “*This seems like an obviously important component to sustainable food systems*” (Participant 10) and “*This characteristic captures the point of sustainable food systems by guaranteeing that we are not doing anything in our day-to-day that, in the long-term, we cannot continue*” (Participant 28). Additionally, among all narratives, the characteristic of democratic and transparent governance is given the most emphasis statistically (by weight of z-score), and it is not seen as unimportant in any narrative.

Here were also three principles that none of the narratives prioritized: producing a substantial amount of food through urban agriculture models (Statement 22), limiting the use of GMOs (Statement 23), and valuing and contributing to the preservation of food traditions and cultures (Statement 33). The idea of limiting GMOs is challenged to some degree by all narratives.

## Discussion

### Understanding perceptions of food system sustainability

Our analysis identified five distinct narratives. These different understandings of sustainable food systems have some common components, which could serve as bridging elements, but also unique characteristics that should be considered when developing action plans for fostering sustainable food systems. By exploring the characteristics important for defining sustainable food systems across all narratives, some shared principles can be established. The consensus around the central importance of food security is notable, given that over 90 percent of consumers have seen higher food prices, and 50 percent have faced food access issues due to climate change, war, the COVID-19 pandemic, and other worldwide events (26). Fairness, justice, and equality are also underscored, as reflected in the consensus across all narratives that the food system should operate under democratically decided rules and norms. However, how these values are manifested differs across narratives.

Interestingly, there is more agreement about what is not a priority for sustainable food systems than on what defines the transformation itself. Among the characteristics not prioritized, it is notable that these are issues over which there is frequent public debate around food systems, society and health, as well as with established regulations, such as the use GMOs in agriculture (27, 28). While the contributions to food security and climate adaptation by crop improvements through genetic engineering may be evident in certain (but not all) contexts, this matter represents a trade-off between environmental and social outcomes related to food systems. Additionally, within the food sovereignty movement, GMOs are increasingly seen as a product of agri-food’s power concentration problem (29). Something similar happens with the importance of urban areas in food systems, and in particular of urban agriculture in the context of sustainable development. This concept, though long present in public policy discussions (30, 31), was not particularly highlighted by any narrative. Similarly, while the preservation of food traditions and cultures, like millets in India (32), is certainly priority among indigenous communities and in the food sovereignty movement (33), this particular facet of food sovereignty was less evident across the identified narratives. It is perhaps notable to emphasize how these general trends reflect the views and concerns of a very particular group of people situated in the context of higher education at a European university, and how the elements reflected in these narratives would likely diverge from those we would find in other social-ecological contexts. In fact, these differences within the rather narrow sample of 31 student participants for this study likely indicate even greater differences across societal groups and point to the need to consider viewpoints from participants involved in the food system from other perspectives in future studies. Clarifying the distinctive perspectives that arise from a larger variety of participants would contribute significantly to the discussion of how to define food systems and how to shape policy around transformation toward sustainable outcomes.

A few Q-statements in this study related to plant-based production and consumption were seen as important at a food system level, but less so when referring to individual behavior, such as dietary changes. This may indicate that animal welfare and plant-based transitions are important overall, but that the personal implications of those transitions may feel less feasible for some (34, 35). Similarly, an emphasis in the literature on sustainable food systems is agroecology (36), but our results indicate that all characteristics of agroecology are not given similar relative importance and may even vary within a single narrative. This may be due to limited familiarity with the concept or the use of alternative terminology outside academic or practitioner contexts. The need for well-recognized and commonly understood terminology among many stakeholders in the food system echoes the already-established importance of wide resonance and interdisciplinary approaches (2, 7).

Notably, environmental concerns are mostly attributed mid-range importance, whereas the statements that specifically relate to methods of production and patterns of consumption generated more divergence. These food-system specific elements may seem more tangible or relatable from the individual’s perspective than global environmental issues associated with general planetary health. Thus, while maintaining planetary boundaries is somewhat agreed upon to be relevant, the linkage between this crucial global target and the food system is not very established, and does not feature as a top priority by the participants in this study.

Our results from this study show the importance of prioritizing sustainable fish stocks, which is the SDG that remains most distant from its target and continues in a negative trend (13). This has promising potential positive implications to biodiversity conservation in aquatic and coastal ecosystems as well as to livelihoods in small-scale fisheries, and a sustainable ocean economy relies upon political issues like stable funding and removal of harmful subsidies (37, 38). The Food and Agriculture Organization (FAO) addresses this through its Blue Transformation program, which promotes aquaculture to adapt to declining global fish stocks and growing food insecurity (8).

### Leverage points for food system transformations

The pathways to achieve food systems sustainability differ across narratives, but some characteristics of sustainable food systems emerge as deep leverage points. These include reducing power concentration in the food value chain, avoiding spillover effects, fostering transparent, democratic governance, and strengthening the human-nature connection. While each perspective comes to similar conclusions about what a sustainable food system’s outcome should be, those outcomes are perceived to manifest differently.

Given the heavy burden that the global food system imposes on other sectorss (39), and the general agreement that challenging corporate concentration could be a lever for greater sustainability, our analysis suggests this issue should be a transformation priority. One approach presented in the literature involves leveraging retailer influence on supply-side externalities (40). However, concrete measures remain limited, as several actors contend that consumer demand for sustainable products is needed first (41). This suggests the need to focus not only on reducing power imbalances but also on creating the conditions, such as transparency, democracy, and consumer empowerment, that make sustainable transformation possible.

## Conclusion

In this study we aimed to identify the diverse narratives university students enrolled in sustainability-related programs regarding the characteristics and priorities of sustainable food system transformations. By focusing on this demographic, the research sought to advance our understanding of the perspectives from the next generation of professionals poised to advance sustainability goals. Our analysis revealed five distinct narratives which converge on the shared principles of long-term food security and transparent, democratic governance. The lack of prioritization for urban agriculture, GMOs, and food traditions suggests context-specific perspectives, which may differ in other social-ecological contexts.

Collectively, the priorities expressed in these narratives can be used to inform teaching practices, program offerings, and institutional policies in the continued ambition toward shaping and participating in sustainable food system transformation. Considering these narratives and where there was consensus and difference, two salient messages conclude this investigation: (1) Addressing the power imbalances of the food system is important to establishing positive outcomes for human and planetary health, for people’s livelihoods, and for choices and preferences about food and lifestyle; and (2) All people have an opinion on sustainable food systems and considering those different viewpoints and their underlying values enables acceptance of pathways toward transformation. To achieve this, several key actions can be taken. Efforts should focus on promoting transparent and democratic governance through inclusive platforms where all citizens and food system actors can actively participate in shaping food systems. This process should be accompanied by empowering consumers with clear information about the environmental and social impacts of alternative choices. Considering these different viewpoints and their underlying values is key to enabling the acceptance of pathways toward transformation.

## Data Availability

The original contributions presented in the study are included in the article/Supplementary material, further inquiries can be directed to the corresponding author.
