# Supplementary material for: Disentangling food transformation narratives: a Q-method analysis on pathways to sustainable food systems and their implications
Source: Front Nutr. 2025 Sep 10;12:1662085. doi: 10.3389/fnut.2025.1662085 (PMC12459661; doi:10.3389/fnut.2025.1662085)
Supplement: Supplementary file 1 [file Supplementary_file_1.docx]

Supplementary material 1. Q-set statements with related concepts. Full reference to sources mentioned follow table.

| **Code** | **Statement: Sustainable food systems…** | **Concept** | **Source** |
| --- | --- | --- | --- |
| 1 | Operate in a manner that ensures that food will still be produced in the future. | Long term food security | Ericksen, 2008 |
| 2 | Allow people to make their own decisions about how to consume and produce food. | Enhancing the concept of food security through agency | HLPE, 2020 |
| 3 | Place power within the food system in the hands of producers and consumers. | Ensuring people's capacity to participate in food system governance | Wahbeh et al., 2022 |
| 4 | Benefit producers with just incomes contributing to livelihoods. | Social dimension of sustainability | Grillow & Bush, 2019 |
| 5 | Reduce the distance between food providers and consumers. | Localized food systems, as envisioned in food sovereignty framework | Grey & Patel, 2015 |
| 6 | Do not allow food to be commodified. | Decommodification of food system, as envisioned in food sovereignty framework | Nyéléni, 2007 |
| 7 | Are guided by rules and norms that are decided democratically and with transparency. | Transforming governance of the food system | Canfield, Duncan & Claeys, 2021 |
| 8 | Continue producing sufficient, nutritious food regardless of disruptions or shocks (i.e., droughts, conflicts, etc.). | Concept of reslience applied to food systems | Tendall et al., 2015 |
| 9 | Provide working conditions that do not harm the mental or physical health of any actor in the food value chain. | Improving working conditions of producers in food value chain | Giordano et al., 2019 |
| 10 | Produce food without synthetic products (i.e., pesticides, herbicides, fertilizers, etc.). | Organic production | Reganold & Wachter, 2016 |
| 11 | Reconcile land management and local communities with natural processes for the common benefit of nature and people. | Agroecological production | Ewert et al., 2023 |
| 12 | Use and protect locally adapted agrobiodiversity (i.e., grains, pulses, vegetable and fruit varieties, animal breeds, etc.). | Ensuring biological diversity in agricultural landscapes | Ewert et al., 2023 |
| 13 | Provide healthy, happy lives for animals. | Protecting animal welfare in animal agriculture | Bryant et al., 2024 |
| 14 | Preserve the quality of food without too much processing. | Addressing ultra-processed food and related health outcomes | Swinburn et al., 2019 |
| 15 | Limit the use of genetically modified organisms (GMOs). | Ensuring biological diversity in agricultural landscapes | National Academy of Sciences, Engineering, and Medicine, 2016 |
| 16 | Ensure the presence of multiple competing actors in every step of the value chain with little concentration of control by a small number of big firms. | Breaking up concentration in the food value chain | Clapp, 2020 |
| 17 | Prioritize local and regional markets. | Localized food systems, as envisioned in food sovereignty framework | Wittman, 2011 |
| 18 | Operate under trading terms that preserve dignity and fairness between parties in the Global North and South. | Addresing food system distribution and trade issues to create a sustainable global market | Clapp, 2020 |
| 19 | Use the minimum needed safe packing materials that do not contribute to waste management problems. | Consumption and waste patterns related to the food system | Westra, 2019 |
| 20 | Clearly and accurately label products on the market. | Improving communication and labeling in retail sector | Macfadyen et al., 2016 |
| 21 | Ensure diets are diverse and nutritious. | Diet transitions needed for sustainable food system outcomes | Swinburn et al., 2019 |
| 22 | Focus on plant-based food production and consumption. | SDGs: 12 / Lancet Recommendations: Halve animal product consumption | Springmann et al., 2019 |
| 23 | Do not contribute to altering biogeochemical cycles (e.g. carbon, nitrogen, water). | SDGs: 13 | Aiking & Erisman, 2021 |
| 24 | Maintain stable populations of fish and aquatic life through sustainable fishing practices. | SDGs: 14 | Independent Group of Scientists, 2023 |
| 25 | Contribute to biodiversity conservation by providing habitat for multiple species. | SDGs: 15 | Foley et al., 2005 |
| 26 | Have neutral CO_2_ emissions. | Remaining within planetary boundaries for the food system | Rockström et al., 2009 |
| 27 | Bring people and nature closer together. |  | Barragan-Jason et al., 2021 |
| 28 | Enrich rural areas so they are lively, healthy communities that add value to society. | Social dimension of sustainability | United Nations, 2019 |
| 29 | Are characterized by innovation, seeking opportunities to improve sustainability in food production, processing, and retailing. | Transforming the political economy of food systems: "Diversity and Innovation" | Canales & Fears, 2023 |
| 30 | Learn from the past and maintain (or reintroduce) sustainable traditional management practices. | Traditional sustainable landscape management | Teil & Lardon, 2022 |
| 31 | Avoid negative impacts on other sectors (i.e., contribute to poor health outcomes or environmental pollution problems, deplete natural resources, etc.). | Transforming the political economy of food systems: "The food-health nexus" | Duncan et al., 2019 |
| 32 | Make education and awareness about nutrition available to everyone. | Transforming the political economy of food systems: "Politics of consumption" | Duncan et al., 2019 |
| 33 | Value and contribute to the preservation of food traditions and cultures. | Transforming the political economy of food systems: "Food sovereignty and Agroecology" | Fakhri, 2024 |
| 34 | Are managed efficiently so that little food is lost from the field to the market. | Lancet Recommendations: Halve food loss | Alexander et al., 2017 |
| 35 | Contain circular systems in which household/consumer food waste is managed responsibly. | Lancet Recommendations: Halve food waste | Cesaro et al., 2022 |
| 36 | Support diets that are dominated by fruits and vegetables. | Lancet Recommendations: Double fruit and vegetable consumption | Willett et al., 2019 |
| 37 | Produce a substantial amount of food through urban agriculture models. | Addressing food system challenges in increasingly urbanized societies | Hebinck & Page, 2017 |

References:

Aiking, H & Erisman, J. W. (2021). Reversing the nitrogen crisis: Role of plant-based diets and sustainable farming. *Alpro Foundation*.

Alexander, P., Brown, C., Arneth, A., Finnigan, J., Moran, D., & Rounsevell, M. D. A. (2017). Losses, inefficiencies and waste in the global food system. *Agricultural Systems*, *153*:190-200. <http://dx.doi.org/10.1016/j.agsy.2017.01.014>

Barragan-Jason, G., de Mazancourt, C., Parmesan, C., Singer, M. C., & Loreau, M. (2021). Human-nature connectedness as a pathway to sustainability: A global meta-analysis. *Conservation Letters*, *15*(1). <https://doi.org/10.1111/conl.12852>

Bryant, C., Hopwood, C. J., Graça, J., Nissen, A. T., Dillard, C., & Thompkins, A. (2024). Exploring public support for farmed animal welfare policy and advocacy across 23 countries. *Psychology of Human-Animal Intergroup Relations*, *3*(e10337). <https://doi.org/10.5964/phair.10337>

Canales, C. & Fears, R. (2023). The role of science, technology, and innovation for transforming food systems in Europe. In von Braun, J. et al. (Eds.), *Science and Innovations for Food Systems Transformation*. <Https://doi.org/10.1007/978-3-031-15703-5_40>

Canfield, M. C., Duncan, J. & Claeys, P. (2021). Reconfiguring food systems governance: The UNFSS and the battle over authority and legitimacy. *Development*, *64*: 181-191. <https://doi.org/10.1057/s41302-021-00312-1>

Cesaro, J-D., Duteurtre, G., Guilbert, S., & Zakhia-Rozis, N. (2022). Urban food waste: A resource for circular economy between cities and agriculture. In Thomas, A. et al. (Eds.), *Sustainable food systems for food security*. Quae.

Clapp, J. 2020. *Food (3rd ed.)*. Medford, MA. Polity Press.

Duncan, J., Levkoe, C. & Moragues-Faus, A. (2019). Envisioning new horizons for the political economy of sustainable food systems. *IDS Bulletin*, *50*(2). <https://doi.org/10.19088/1968-2019.117>

Ericksen, P. J. 2008. “Conceptualizing Food Systems for Global Environmental Change Research.” *Global Environmental Change* 18 (1): 234–45. <https://doi.org/10.1016/j.gloenvcha.2007.09.002>.

Ewert, F., R. Baatz, and R. Finger. 2023. “Agroecology for a Sustainable Agriculture and Food System: From Local Solutions to Large-Scale Adoption.” *Annual Review of Resource Economics* 15 (1): 351–81. <https://doi.org/10.1146/annurev-resource-102422-090105>.

Fakhri, M. (2024a). *The right to food, violence, and food systems*. Asser Press.

Foley, J. A., DeFries, R., Asner, G. P., Barford, C., Bonan, G., Carpenter, S. R., Chapin, F. S., Coe, M. T., Daily, G. C., Gibbs, H. K., Helkowski, J. H., Holloway, T., Howard, E. A., Kucharik, C. J., Monfreda, C., Patz, J. A., Pretice, I. C., Ramankutty, N., & Snyder, P. K. (2005). Global consequences of land use. *Science*, *309*(5734):570-574. <https://doi.org/10.1126/science.1111772>

Giordano, T., Losch, B., Souriseau, J.-M., & Girard, P. (2019). Risks of mass unemployment and worsening of working conditions. In Dury, S. et al. (Eds.), *Food systems at risk: New trends and challenges*. FAO, CIRAD.

Grey, S. and R. Patel. 2015. “Food Sovereignty as Decolonization: Some Contributions from Indigenous Movements to Food System and Development Politics.” *Agriculture and Human Values* 32 (3): 431–44. <https://doi.org/10.1007/s10460-014-9548-9>.

Grillow, J. & Bush, J. (2019). Applying the household economy analysis to measure and address income gaps in agriculture supply chains. *The Living Income Community of Practice*. <https://www.living-income.com/fileadmin/living_income/Publications/Actual_Income_and_Gap_Measurement/FINAL_HEA_and_Living_Income_Benchmarks_guidance_doc_.pdf>

Hebinck, A., & Page, D. (2017). Processes of Participation in the Development of Urban Food Strategies: A Comparative Assessment of Exeter and Eindhoven. *Sustainability*, *9*(6), Article 931. <https://doi.org/10.3390/su9060931>

High Level Panel of Experts on Food Security and Nutrition (HLPE). (2020). *Food security and nutrition: Building a global narrative towards 2030*. Rome.

Independent Group of Scientists appointed by the Secretary-General. 2023. *Global Sustainable Development Report 2023: Times of crisis, times of change: Science for accelerating transformations to sustainable development*. United Nations*.* <https://sdgs.un.org/gsdr/gsdr2023>

Macfadyen, S., J. M. Tylianakis, D. K. Letourneau, T. G. Benton, P. Tittonell, M. P. Perring, C. Gómez-Creutzberg, et al. 2016. “The Role of Food Retailers in Improving Resilience in Global Food Supply.” *Global Food Security* 7 (December):1–8. <https://doi.org/10.1016/j.gfs.2016.01.001>.

National Academy of Sciences, Engineering, and Medicine. (2016). Genetically Engineered Crops: Experiences and prospects. *The National Academies Press*. <https://doi.org/10.17226/23395>.

Nyéléni Forum for Food Sovereignty. (2007). *Declaration of the Forum for Food Sovereignty*. Selingué, Mali, 23–27 February.

Reganold, J. P. & Wachter, J. M. (2016). Organic agriculture in the twenty-first century. *Nature Plants*, *2*(15221). <https://doi.org/10.1038/nplants.2015.221>

Rockström, J., W. Steffan, K. Noone, Å. Persson, F. S. Chapin III, E. F. Lambin, T. M. Lenton, et al. 2009. “A Safe Operating Space for Humanity. *Nature* 461: 472–475. <https://doi.org/10.1038/461472a>

Shiva, V. (2024, April 6). GMO Bio-Imperialism. *Navdanya International*. <https://navdanyainternational.org/gmo-bio-imperialism/>

Springmann, M., M. Clark, D. Mason-D’Croz, K. Wiebe, B. L. Bodirsky, L. Lassaletta, W. de Vries, et al. 2018. Options for Keeping the Food System within Environmental Limits. *Nature* 562: 519-525. <https://doi.org/10.1038/s41586-018-0594-0>

Swinburn, B. A., V. I. Kraak, S. Allender, V. J. Atkins, P. J. Baker, J. R. Bogard, H. Brinsden, et al. 2019. “The Global Syndemic of Obesity, Undernutrition, and Climate Change: The Lancet Commission Report.” *The Lancet* 393 (10173): 791–846. <https://doi.org/10.1016/S0140-6736(18)32822-8>.

Teil, G. & Lardon, S. (2022). Neither quite the same nor quite another: Diversity, identity and resilience in agroecology. In Thomas, A. et al. (Eds.), *Sustainable food systems for food security*. Quae.

Tendall, D. M., Joerin, J., Kopainsky, B., Edwards, P., Shreck, A., Le, Q. B., Kruetli, P., Grand, M., & Six, J. (2015). Food system resilience: Defining the concept. *Global Food Security*, *6*:17-23. <http://doi.org/10.1016/j.gfs.2015.08.001>

United Nations, Department of Economic and Social Affairs, Population Division. (2019). *World Urbanization Prospects: The 2018 Revision (ST/ESA/SER.A/420)*. New York: United Nations.

Wahbeh, S., Anastasiadis, F., Sundarakani, B., Manikas, I. (2022). Exploration of food security challenges towards more sustainable food production: A systematic literature review of the major drivers and policies. In Raposo, A. et al. (Eds.), *Challenging the status quo to shape food systems transformation from a nutritional and food security perspective* (2nd ed.). MDPI.

Westra, E. H. (2019, November 18). The packaging of food: How to minimalise environmental impact. *Wageningen University & Research*. <https://www.wur.nl/en/show-longread/the-packaging-of-food-how-to-minimalise-environmental-impact-f006pa.htm>

Willett, W., J. Rockström, B. Loken, M. Springmann, T. Lang, S. Vermeulen, T. Garnett, et al. 2019. “Food in the Anthropocene: The EAT–Lancet Commission on Healthy Diets from Sustainable Food Systems.” *The Lancet* 393 (10170): 447–92. <https://doi.org/10.1016/S0140-6736(18)31788-4>

Wittman, H. 2011. “Food Sovereignty: A New Rights Framework for Food and Nature?” *Environment and Society* 2 (1). <https://doi.org/10.3167/ares.2011.020106>.

Supplementary material 2. Characteristics of participants with indication of the mode of participation, to which factor (if any) they were associated (flagged), demographic and background information. The first and last priorities of each participant are noted as well as if their contextual responses contained a reference thematically tied to leverage points theory. If a participant did not respond to the follow-up questionnaire, “no response” or “N/A” are noted in the columns where there was no data to reference.

| **Participant** | **Mode of Participation** | **Associated to Which Factor** | **Factor Loadings (threshold of 0.32)** | **Age** | **Gender** | **Cultural Background** | **Field of Study/Practice** | **Dietary Preference** | **Buys Groceries At…** | **First priority** | **Last priority** |
| --- | --- | --- | --- | --- | --- | --- | --- | --- | --- | --- | --- |
| P1 | online | Not flagged | F1 0.5645, F2 0.388 | 18-24 | Woman | French/British | Environment and Resource Management, Food Systems | Vegan | Supermarket | S16 | S5 |
| P2 | in person | F1 | 0.8204 | 35-44 | Woman | North American | Environment and Resource Management, Food Systems | Vegetarian | Supermarket, Organic Supermarket, Local Market, and Direct Farm Purchasing. | S16 | S37 |
| P3 | in person | F2 | 0.762 | 18-24 | Man | Swiss | Chemistry | None | Supermarket | S1 | S15 |
| P4 | in person | F2 | 0.704 | 18-24 | Man | Dutch | Chemistry | None | Supermarket | S1 | S15 |
| P5 | online | F1 | 0.7784 | 25-34 | Woman | South European | Environmental Studies | No Response | Supermarket | S11 | S15 |
| P6 | online | Not Flagged | F2 0.504, F4 0.63149 | 18-24 | Man | Moroccan | Biomedical Sciences | Halal | Supermarket and Local Markets/Butchers | S1 | S22 |
| P7 | online | F4 | 0.81034 | 18-24 | Man | Serbian | Biomedical Sciences | No Response | No Response | S1 | S15 |
| P8 | in person | F5 | 0.64013 | 25-34 | Man | American | Data Sciences | None | Supermarket | S7 | S15 |
| P9 | in person | F2 | 0.525 | 25-34 | Woman | Dutch/North African | Data Sciences | None | Supermarket, Cultural Food Stores, and Local Market | S20 | S8 |
| P10 | in person | F2 | 0.718 | 25-34 | Man | Indian | Energy Transition | Vegetarian | Supermarket, Cultural Food Stores, and Direct Farm Purchasing | S1 | S15 |
| P11 | online | F4 | 0.38395 | No Response | Man | No Response | No Response | No Response | No Response | S15 | S22 |
| P12 | in person | Not Flagged | F3 0.5096, F5 0.33332 | 18-24 | Man | Dutch | Philosophy, Politics, Economics | None | Supermarket | S31 | S33 |
| P13 | in person | F5 | 0.52603 | 18-24 | Man | Dutch | Philosophy, Politics, Economics | None | Supermarket and Organic Supermarkets | S32 | S33 |
| P14 | in person | F3 | 0.6729 | 18-24 | Man | Danish/Bosnian | Law | Vegetarian | Supermarket and Local Markets | S14 | S28 |
| P15 | in person | F3 | 0.7369 | 25-34 | Woman | Dutch | Computer Science | Vegetarian | Supermarket | S13 | S33 |
| P16 | in person | F2 | 0.334 | 25-34 | Man | German | Computer Science | None | Supermarket | S27 | S2 |
| P17 | in person | F2 | 0.612 | 18-24 | Man | European/Asian | Environment and Resource Management, Food Systems | None | Supermarket and Local Market | S31 | S28 |
| P18 | in person | F3 | 0.5419 | 18-24 | Woman | Polish | Environment and Resource Management | None | Supermarket | S26 | S28 |
| P19 | in person | F5 | 0.61068 | 18-24 | Woman | Dutch | Environmental Science | Vegan | Supermarket | S7 | S2 |
| P20 | online | F4 | 0.53414 | 18-24 | Man | Dutch | Geology | No Response | No Response | S1 | S13 |
| P21 | in person | F4 | 0.59350 | 18-24 | Woman | Dutch/Russian | No Response | None | Supermarket | S2 | S15 |
| P22 | online | F2 | 0.635 | No Response | Man | Dutch | No Response | No Response | No Response | S1 | S7 |
| P23 | online | F1 | 0.8310 | 18-24 | Woman | American | Biology | Vegetarian | Supermarket | S8 | S37 |
| P24 | online | F3 | 0.6457 | 25-34 | Woman | Dutch | Social Science | Vegetarian | Supermarket | S31 | S27 |
| P25 | online | F4 | 0.39886 | 18-24 | Woman | Indian/Dutch | Law | Vegetarian | Supermarket and Organic Supermarket | S14 | S27 |
| P26 | online | F3 | 0.6484 | 18-24 | Woman | Hungarian | Cultural Anthropology | None | Supermarket and Food-Saving Programs | S34 | S6 |
| P27 | online | F4 | 0.62970 | 18-24 | Man | Dutch | Health Sciences | No Response | No Response | S1 | S37 |
| P28 | online | F1 | 0.7663 | 25-34 | Man | Dutch | Food Systems | Vegetarian | Organic Cooperative Supermarket | S1 | S37 |
| P29 | online | F1 | 0.7797 | 25-34 | Woman | No Response | Community Organizing, Food Systems | None | Supermarket and Grows Own Food | S8 | S29 |
| P30 | online | F1 | 0.6711 | 25-34 | Woman | Dutch | Social Sciences, Sustainability transitions | Vegetarian | Supermarket | S27 | S37 |
| P31 | online | F5 | 0.63306 | 25-34 | Man | European | Food Systems | Vegetarian | Farmers Markets and Package Free Stores | S27 | S29 |
